# Efficacy of Local Autologous Platelet-Rich Plasma in the Treatment of Pressure Ulcer in Spinal Cord Injury Patients

**DOI:** 10.7759/cureus.18668

**Published:** 2021-10-11

**Authors:** Gurpreet Singh, Diganta Borah, Geetika Khanna, Sakshi Jain

**Affiliations:** 1 Physical Medicine and Rehabilitation, Vardhman Mahavir Medical College and Safdarjung Hospital, New Delhi, IND; 2 Pathology, Vardhman Mahavir Medical College and Safdarjung Hospital, New Delhi, IND; 3 Physical Medicine and Rehabilitation, Atal Bihari Vajpayee Institute of Medical Sciences and Dr. Ram Manohar Lohia Hospital, New Delhi, IND

**Keywords:** platelet-rich plasma, ulcer healing, hydrogel dressing, pressure ulcer, spinal cord injury

## Abstract

Background: Pressure ulcer is one of the common complications occurring in spinal cord injury (SCI) patients. Platelet-rich plasma (PRP) has been found useful in the treatment of pressure ulcers in few studies. The purpose of this study was to evaluate the role of PRP in pressure ulcer healing in comparison to hydrogel dressing in SCI patients.

Methods: In this randomized interventional study, 52 patients of SCI having pressure ulcers of grade III/IV were randomized into two groups of 26 each. In group A patients, hydrogel dressing was done while freshly prepared PRP was used in patients of group B. Pressure ulcers were evaluated at baseline and after three weeks and six weeks in terms of ulcer surface area, volume, Pressure Ulcer Scale for Healing (PUSH) score, histopathology, and ulcer healing parameters. Data were collected and quantitative variables were compared using unpaired t-test or Mann-Whitney test between the two groups and qualitative variables were compared using the chi-square test or Fisher’s exact test. A p-value of <0.05 was considered statistically significant.

Results: Baseline characteristics were comparable in both groups. There was a significant improvement in ulcers in terms of surface area, volume, and PUSH score in both the groups but it was comparable (p-value >0.05). There was a significant improvement in the PRP group as compared to the other group in terms of epithelization, granulation, and neovascularization at three and six-week follow-up.

Conclusions: This study suggests that PRP is a possible and better alternative to conventional dressing methods for the treatment of pressure ulcers.

## Introduction

Spinal cord injury (SCI) is a catastrophic event in one’s life, resulting in varying degrees of disability depending on the level and severity of the lesion. Globally, the incidence varies from 13 to 220 per million population [1]. A considerable proportion of SCI patients develop one or other complications such as pneumonia, orthostatic hypotension, autonomic dysreflexia, urinary tract infection, constipation, osteoporosis, and heterotopic ossification during their lifetime. Pressure ulcer (PrU) is one such complication occurring in about one in three patients with SCI [2]. PrU is defined as a localized injury to the skin and/or underlying tissue usually over bony prominences, as a result of pressure or pressure in combination with shear [3].

PrU in SCI patients rewrites the life of the patient by adversely affecting the medical condition as well as the overall rehabilitation of the patients. Its negative impact on health and quality of life has been well documented in the literature [4-6]. Moreover, it imposes an increased economic burden on the family by increasing the cost of healthcare and living. Stroupe et al. reported about 2.6 times higher annual treatment costs and higher healthcare utilization in SCI patients with PrU in comparison to those without PrU [7]. Researchers have indicated the association of PrU with increased morbidity and mortality in SCI patients [8-10]. Therefore, effective treatment can provide a substantial change in the overall rehabilitation of the patient.

Management of PrU involves wound care, management of contributory risk factors, and other general measures. Wound care includes debridement and cleansing, treatment of infection if any, and dressing. Various dressing materials have been used including hydrocolloid dressing, hydrogel dressing, impregnated gauze dressing, alginate dressing, etc. Other wound care modalities including negative pressure wound therapy, surgical management, autologous platelet-derived growth factors, and adjunctive therapies like electrical stimulation, hydrotherapy, hyperbaric oxygen, and autologous platelet-rich plasma (PRP) are also in practice. To the best of our knowledge, literature is inconclusive about which dressing material/ technique is best. In recent times, the use of PRP in the treatment of various chronic ulcers including diabetic foot ulcers and pressure ulcers is gaining momentum. In chronic ulcers, the natural process of wound healing is deranged and characterized by an abnormally prolonged inflammatory phase [11]. Therefore, for an intervention to be effective, it must modify the environment to shorten the inflammatory phase and induce the reparative phase [11]. The crucial role of platelets in wound healing supports the application of platelet concentrates such as PRP for treatment of chronic wound [11]. Autologous platelet-derived wound-healing factors have been proposed to regulate wound healing of chronic cutaneous ulcers by promoting the formation of granulation tissue in the early healing phase. Researchers reported the usefulness of PRP in the treatment of chronic ulcers including pressure ulcer [12-18]. However, further studies have been suggested for consolidation of these findings [12,16-18]. Also, most of the previous studies assessed the effect of PRP on two-dimensional characteristics, assessing the area of the ulcer and clinical improvements. We propose better healing of PrU following treatment with local PRP application. Therefore, this study was conducted to assess the effect of local PRP in the treatment of PrU in comparison to hydrogel dressing, which is the current practice in our institute, using a wide range of assessment parameters including volumetric and histopathological changes.

## Materials and methods

This prospective study was conducted in the Department of Physical Medicine and Rehabilitation of a tertiary care hospital in India from October 2017 to March 2019. The study was undertaken after the approval of the Institutional Ethical Committee (IEC - Oct/2017-178). Spinal cord injury patients below C6 level with grade 3 or 4 sacral pressure ulcer according to National Pressure Ulcer Advisory Panel (NPUAP) scale [3] were included in the study. Patients with bleeding/clotting disorder, thrombocytopenia, or malignancy, or patients on immunosuppressant/antiretroviral drugs were excluded from the study. After obtaining written informed consent, all diagnosed cases of SCI were examined and screened according to inclusion and exclusion criteria. A total of 52 patients were enrolled in the study. Complete workup of all the participants was done including a detailed history, clinical examination, and relevant investigations.

Patients were randomized into two groups (A and B) by sealed envelope randomization, with 26 patients in each group. In all patients, the ulcer was cleaned with normal saline, and debridement was done if required. In group A patients, the hydrogel was applied all over the wound and covered with sterile cotton gauze, while in group B patients, freshly prepared PRP was injected into the ulcer margin and base, and applied over the ulcer, and covered with the sterile cotton gauze. Dressing of the ulcer was done twice a week for six weeks in the same technique in patients of respective groups. The endpoint of the study was marked by healed ulcer/completion of six weeks of intervention, patient not willing to continue in the study, or deterioration in terms of surface area/necrotic tissue/discharge.

Each patient was assessed before the intervention and after three weeks and six weeks of the start of intervention using predefined assessment tools. The surface area of the ulcer was assessed by mapping the ulcer on a transparent sheet and measuring the area with the help of graph paper. The volume of the pressure ulcer was measured by completely filling the ulcer crater with sterile normal saline from a graduated syringe without overflowing the saline. For histopathological examination, a punch biopsy was taken from the margin of the wound. Histopathological examination was carried out in the pathology laboratory of the same hospital. Granulation tissue, neovascularization, epithelization, and necrosis in tissue were assessed and graded as grade 0 to 3 in a semiquantitative grading: grade 0 - absent, grade 1 - scarcely present, grade 2 - present, and grade 3 - intensively present [19]. Healing of the pressure ulcer was assessed using Pressure Ulcer Scale for Healing (PUSH), percentage of healed ulcer area, and healing rate. PUSH comprises three sub-scores, assessing ulcer surface area, amount of exudate, and tissue type. Minimum and maximum possible scores for PUSH are 0 and 17, respectively [20]. Percentage of healed ulcer area and healing rate were assessed using the method used by ElHeneidy et al. in their study on wound healing [21].

Statistical analysis was done using Statistical Package for the Social Sciences (SPSS) version 21 (IBM Corp., Armonk, NY). Categorical variables were presented in number and percentage and continuous variables were presented as mean ± SD. Normality of data was tested by Kolmogorov-Smirnov test. If the normality was rejected then a non-parametric test was used. Quantitative variables were compared using unpaired t-test/Mann-Whitney test (when the datasets were not normally distributed) between the two groups and qualitative variables were compared using chi-square test/Fisher’s exact test. A p-value of <0.05 was considered statistically significant.

## Results

A total of 52 patients enrolled in the study. Baseline characteristics of the patients of the two groups are depicted in Table 1. Out of 52 patients, 43 were male and nine were female, and they were evenly distributed between the two groups. Two patients in group A did not continue the study after the initial assessment and intervention. Most of the patients had SCI due to trauma like road traffic accident and fall from height. The enrolled patients were from 18 to 60 years of age and no difference was noted between the two groups in age distribution. The level of injury of the patients ranged from C8 to L1 with comparable distribution over the two groups. Maximum patients (90.38 %) were having grade 4 ulcers in both groups.

**Table 1:** Baseline characteristics of patients in both groups.

Characteristics		Group A	Group B	p-value
Age	≤20 years	4	4	0.778
	21-30 years	7	5	
	31-40 years	6	7	
	41-50 years	5	8	
	51-60 years	4	2	
Gender	Male	20	3	0.465
	Female	6	23	
Level of injury	C8	0	1	0.236
	D5	2	0	
	D6	2	1	
	D7	2	4	
	D8	3	2	
	D9	2	3	
	D10	6	1	
	D11	1	5	
	D12	4	7	
	L1	4	2	
Grade of ulcer	Grade III	4	1	0.350
	Grade IV	22	25	
Surface area of ulcer (mean)		36.38	37.04	0.164
Volume of Ulcer (mean)		8	7.03	0.223

**Figure 1:**
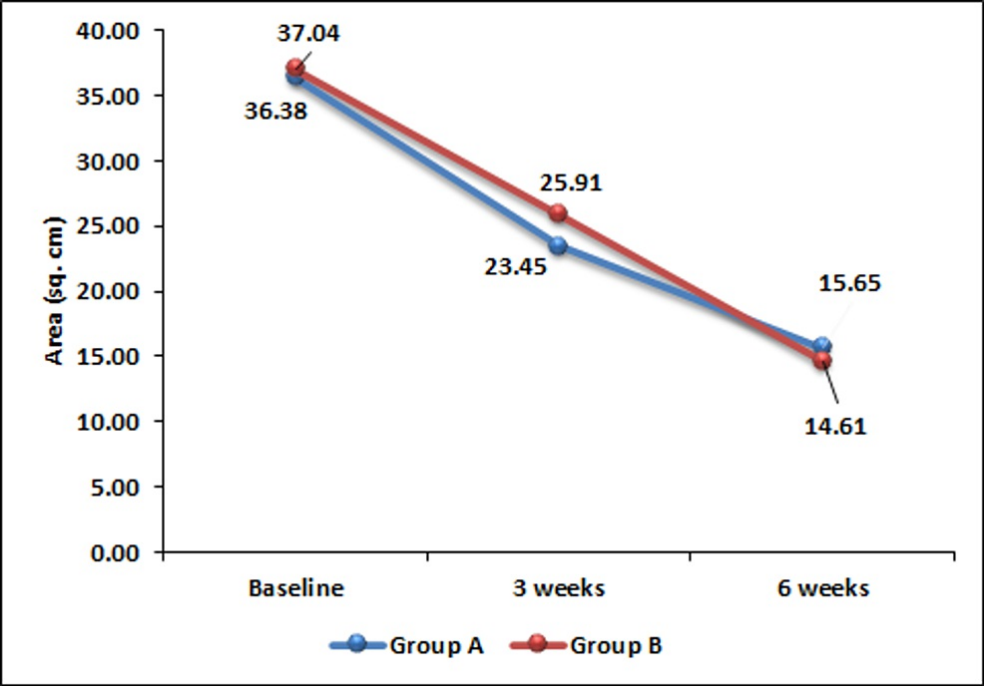
Change in ulcer area over time.

Mean baseline surface areas of 36.38 cm^2^ in group A and 37.04 cm^2^ in group B were found to be reduced to 23.45 cm^2^ and 25.91 cm^2^, respectively, three weeks after initiation of intervention. This change in surface area was statistically significant. Further, a significant reduction of surface area was observed after six weeks also. This indicates the usefulness of both the modalities in reducing the surface area of the ulcer. However, in terms of reduction of surface area, no significant difference was noted between the two treatment modalities over the study period (Figure 1).

**Figure 2:**
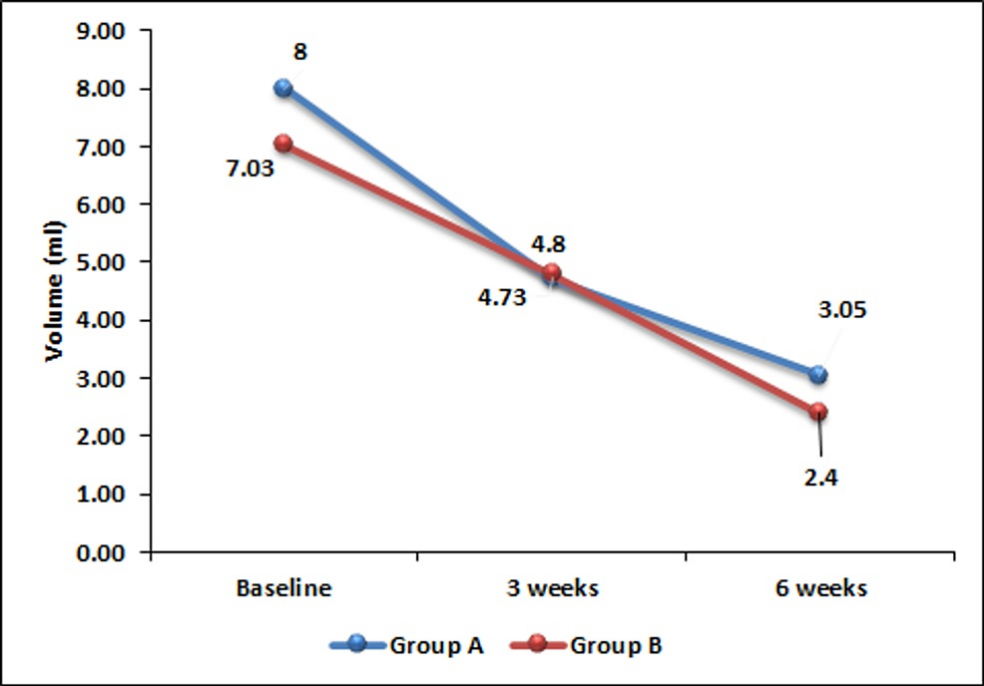
Change in ulcer volume over time.

At the baseline, the mean volume of the ulcer of the two groups were comparable (p = 0.223) and remained comparable throughout the study period. However, both the groups showed a significant decrease in the volume of ulcers at three weeks (p < 0.0001) and six weeks (p < 0.0001) (Figure 2).

Histopathologically, grade 1 granulation tissue was observed in most of the patients (65.38 %) at the initial assessment (Table 2). In respect of the granulation tissue pattern of the ulcer, the patients were evenly distributed between the two groups (p = 0.353). Improvement in the form of change of granulation tissue grading was observed at three weeks following PRP therapy (p = 0.0005). However, non-significant changes were observed in group A patients. Thus, a better result was recorded in favor of PRP (p = 0.002). After six weeks of dressing, most of the patients were having grade 2 granulation tissue without any significant difference between the two groups (p = 0.762).

**Table 2:** Histopathological appearance of the ulcer – granulation tissue.

Granulation grading	Baseline		3 weeks			6 weeks		
	Groups		Groups		p-value	Groups		p-value
	Group A (N = 26)	Group B (N = 26)	Group A (N = 24)	Group B (N = 26)		Group A (N = 24)	Group B (N = 26)	
0	4	3	1	0	Group A = 0.264, Group B = 0.0005	3	2	Group A < 0.0001, Group B = 0.0003
1	1	16	16	5		2	2	
2	4	4	7	18		13	12	
3	0	3	0	3		6	10	
p-value	0.353		0.002			0.762		

Baseline grading of necrosis showed mixed grading in both groups without any statistical significance (p = 0.168). No preponderance to any specific grade was demonstrated. A substantial decrease in necrosis was observed in both the groups at three weeks and six weeks following treatment (Table 3). In the PRP group, out of 26 patients, 25 changed to grade 0 and only one was in grade 1 (p = 0.0002). In the hydrogel group also similar findings were recorded (p < 0.0001). But when compared between the groups, these changes were not statistically significant (p = 1).

**Table 3:** Histopathological appearance of the ulcer – necrosis.

Necrosis grading	Baseline		3 weeks			6 weeks		
	Groups		Groups		p-value	Groups		p-value
	Group A (N = 26)	Group B (N = 26)	Group A (N = 24)	Group B (N = 26)		Group A (N = 24)	Group B (N = 26)	
0	6	9	19	19	Group A = 0.0008, Group B = 0.019	23	25	Group A = 0.0002, Group B < 0.0001
1	10	4	4	5		1	1	
2	8	6	1	1		0	0	
3	2	7	0	1		0	0	
p-value	0.168		0.793			1.000		

At the entry into the study, the two groups were comparable in respect of epithelization (p = 0.492). Overall, grade 0 epithelization was present in 48 patients, grade 1 in three patients, and grade 2 in only one patient (Table 4). Significant epithelization was observed after three weeks of dressing in the PRP group (p < 0.0001) as well as in the hydrogel group (p = 0.001) with the PRP group showing significantly better improvement (p = 0.01). Epithelization after six weeks of dressing showed marked improvement in the PRP group (p < 0.0001). A similar result was recorded in the hydrogel group also (p < 0.0001). In a comparison of the two groups, better improvement was recorded in the PRP group (p < 0.0001).

**Table 4:** Histopathological appearance of the ulcer – epithelization.

Epithelization grading	Baseline		3 weeks			6 weeks		
	Groups		Groups		p-value	Groups		p-value
	Group A (N = 26)	Group B (N = 26)	Group A (N = 24)	Group B (N = 26)		Group A (N = 24)	Group B (N = 26)	
0	25	23	12	4	Group A = 0.001, Group B = <0.0001	3	5	Group A = <0.0001, Group B = <0.0001
1	1	2	10	10		14	1	
2	0	1	2	9		4	2	
3	0	0	0	3		3	18	
p-value	0.492		0.01			<0.0001		

Initial histological assessment of neovascularization revealed that most of the patients were having grade 0 or grade 1 neovascularization (Table 5). Only two patients had grade 3 neovascularization in the PRP group. Patients were evenly distributed over the two groups (p = 0.463). After six weeks of dressing, 15 PRP group patients showed grade 2 neovascularization with only two patients showing grade 0. The change in neovascularization in the PRP group was significant (p = 0.002). On the other hand, the same could not be demonstrated in the hydrogel group (p = 0.067). Thus, neovascularization after treatment with PRP was found to be significantly better as compared to hydrogel at the end of six weeks (p = 0.038).

**Table 5:** Histopathological appearance of the ulcer – neovascularization.

Neovascularization grading	Baseline		3 weeks			6 weeks		
	Groups		Groups		p-value	Groups		p-value
	Group A (N = 26)	Group B (N = 26)	Group A (N = 24)	Group B (N = 26)		Group A (N = 24)	Group B (N = 26)	
0	11	11	2	0	Group A = 0.023, Group B = 0.002	3	2	Group A = 0.067, Group B = 0.002
1	12	9	18	15		13	5	
2	3	4	4	9		7	15	
3	0	2	0	2		1	4	
p value	0.463		0.106			0.038		

**Figure 3:**
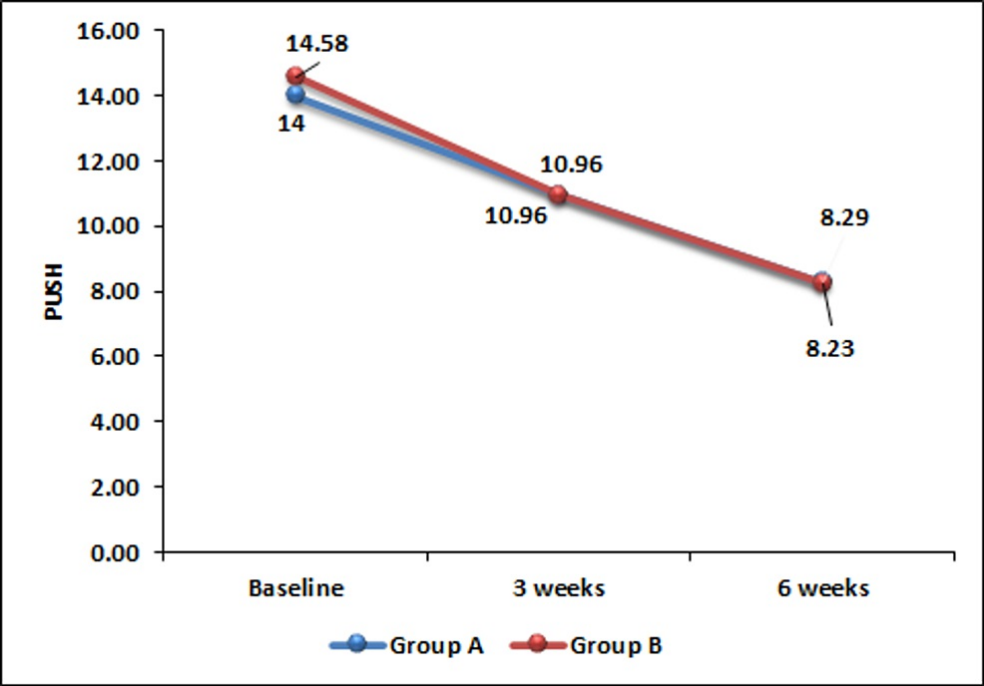
Change in PUSH score over time. PUSH, Pressure Ulcer Scale for Healing.

The Pressure Ulcer Scale for Healing (PUSH) score was found to be improved significantly from baseline to three weeks and six weeks after initiation of intervention in both the groups (Figure 3). However, no significant difference was observed between the two groups indicating comparability of the two interventions.

The percentage of healed ulcer area was measured at three weeks and six weeks after initiation of intervention. A significant increase in this area from three weeks to six weeks was observed in both groups (p < 0.01). While comparing the two groups, a significantly better result was observed in the PRP group at six weeks (p < 0.015).

Healing rate as measured in square cm/week was comparable throughout the study period and no significant difference was recorded between the two groups.

## Discussion

Pressure ulcer in spinal cord injury patients remains a major impediment in rehabilitation as well as in daily life of the patient. Various treatment modalities are in use for its management. Researchers have documented the effectiveness of PRP in the management of chronic wounds including pressure ulcers in SCI patients. This study was conducted for a comprehensive evaluation of efficacy of PRP in the treatment of PrU in SCI patients.

Most of the parameters assessed showed improvement in both groups. However, a significant difference between the two groups was observed in some of the parameters pointing to the superiority of one over the other.

The surface area of the PrU was found to be significantly reduced after three weeks (p < 0.0001) and at six weeks (p < 0.0001) of treatment with PRP as well as hydrogel dressing. Reduction of the surface area of ulcer with PRP dressing has been documented by Singh et al., which was found to be more efficacious than saline dressing [12]. In another study assessing the efficacy of PRP in the treatment of chronic venous leg ulcers, Somani et al. reported a significant reduction of ulcer area in comparison to saline dressing [13]. However, in the present study, comparative efficacy of PRP was noted with hydrogel dressing in terms of reducing the surface area of the ulcer. In contrast to these findings, PRP could not demonstrate an appreciable reduction in surface area of ulcers in a study reported by Tsachiridi et al. [16].

All the patients included in this study showed a significant reduction of ulcer volume as compared to the initial volume. We could not find any difference in efficacy between PRP and hydrogel in reducing the volume of ulcers. Reduction of volume following PRP therapy has been reported in a case series by Sell et al. [15]. However, studies comparing the change in volume of pressure ulcers could not be found in our literature search.

PUSH score reduced significantly following PRP as well as hydrogel dressing. PRP treatment resulted in a better outcome in terms of PUSH score as compared to the hydrogel. This finding of our study is in accordance with the findings reported by Singh et al. showing the superiority of PRP over normal saline dressing [12].

Histopathological findings of the patients showed a non-healing pattern at the beginning of the study. All the ulcers improved to the healing stage following treatment with PRP and also with hydrogel. Similar results were observed with PRP treatment by Singh et al. [12]. In the present study, significant improvement in granulation tissue was observed as early as three weeks in patients treated with PRP, which became more so at six weeks. However, in patients treated with hydrogel, a significant change was noted only at six weeks, indicating better granulation tissue formation with PRP. Clinically appreciable granulation tissue formation following treatment with PRP has been reported by various researchers [15,22-25]. Back in 1986, Knighton et al. demonstrated accelerated growth of granulation tissue and epithelization in chronic non-healing wounds following topical application of autologous platelet-derived wound healing factors [26]. Singh et al. reported histopathological evidence of well-formed granulation tissue and epithelization with PRP treatment in 60% of their patients at fifth week [12]. In the present study, a significant gain in epithelization was recorded. Of enrolled patients, 92% were found to show an absence of epithelization, which started to show improvement at three weeks following treatment and continued to improve till six weeks. Although this improvement was noted in both groups, PRP resulted in a significantly better outcome. Clinical appreciation of epithelization has been recorded in the literature [15,24,26]. The present study showed appreciable vascularization at three weeks and six weeks following treatment. This development was noted to be better in patients treated with PRP. Similar improvement with PRP therapy was recorded earlier by various researchers [12,15]. Also, the angiogenetic property of PRP has been documented by various authors in animal models as well as in humans [12,27-29]. In the present study, PRP demonstrated a better outcome as evidenced by early granulation tissue formation, significantly more epithelization, and neovascularization as compared to dressing with hydrogel.

In gross observation of healing, a significant increase in healing area from the third week to the sixth week following intervention was noted in both groups. Singh et al. reported a significantly better percentage of healing area at the end of treatment with PRP than with normal saline [12]. The final mean percentage of healing area at the end of five weeks was found to be 57.94%. In the present study, the same was recorded as 65.16% at six weeks, which was a significantly better outcome in comparison to hydrogel (p = 0.015). Similarly, Anitua et al. reported a mean percentage of the healed area of 72.94% with PRP at eight weeks in comparison to 21.48% with standard treatment in patients with chronic ulcers of varying etiology [25].

The findings of our study demonstrated the efficacy of PRP in the treatment of pressure ulcers and are supported by previously reported findings by various researchers. However, there are some limitations of the present study. Only sacral pressure ulcers were included in the study. Thus, the effect of the anatomical location of the pressure ulcer could not be commented on. Although the pathologist conducting the histopathological examination was blinded to the applied treatment modality, other investigators were not blinded. The sample size was also limited. Future research in the subject matter should consider these shortcomings of the present study.

## Conclusions

This comprehensive evaluation of the efficacy of PRP in the treatment of pressure ulcers in spinal cord injury patients reaffirms the findings of previous literature in the subject matter. The results of this study show an overall improvement in histopathological and clinical findings including three-dimensional ulcer size, which is possibly the first of its kind. Evidence in the literature and the findings of this study suggest that PRP is a possible and better alternative to conventional dressing methods for the treatment of pressure ulcers. However, a study involving a larger sample size is warranted for further consolidation of the findings of the present study.
